# Financial Burden of General Surgeries by Insurance Status: A Single-Center Case Study from a Quaternary Care Teaching Hospital in Karnataka, India

**DOI:** 10.3390/healthcare14050587

**Published:** 2026-02-26

**Authors:** Rajesh Kamath, Reena Verma, Naaz, Rajib Mandal, Tarushree Bari, Varshini R. Jayapriya, Ashok Kamat, Sagarika Kamath, Anindita Ghosh, Nahima Akthar, Ravichandran Nair, Manjunath Laxminarayana

**Affiliations:** 1 Department of Healthcare and Hospital Management, Prasanna School of Public Health, Manipal Academy of Higher Education, Manipal 576104, India; rajesh.kamath@manipal.edu (R.K.); naaz.psphmpl2023@learner.manipal.edu (N.); varshini.psphmpl2023@learner.manipal.edu (V.R.J.); 2 School of Nutrition and Dietetics, Symbiosis Skills and Professional University, Kiwale, Pune 412101, India; reena.verma@sspu.ac.in (R.V.); anindita.ghosh@sspu.ac.in (A.G.); 3 Programme of Optometry, Faculty of Paramedical Sciences, Assam down town University, Sankar Madhab Path, Gandhinagar, Panikhaiti, Guwahati 781026, India; rajib.mandal@adtu.in; 4 Directorate of Online Education, Manipal Academy of Higher Education, Manipal 576104, India; drtarustats@gmail.com (T.B.); nahima.a@manipal.edu (N.A.); 5 Department of Psychiatric Nursing, Arihant Institute of Nursing Sciences, Belagavi 590010, India; principalarihantins@gmail.com; 6 Department of International Health, Care and Public Health Research Institute—CAPHRI, Faculty of Health, Medicine and Life Sciences, Maastricht University, 6200 MD Maastricht, The Netherlands; dr.sagarikarkamath@gmail.com; 7 Department of Social and Health Innovation, Prasanna School of Public Health, Manipal Academy of Higher Education, Manipal 576104, India; 8 Department of Paediatrics, Kasturba Medical College, Manipal Academy of Higher Education, Manipal 576104, India

**Keywords:** Out-of-Pocket Expenditure (OOPE), Ayushman Bharat Pradhan Mantri Jan Arogya Yojana (AB-PMJAY), private health insurance, uninsured patients, general surgery

## Abstract

Background: Surgical care is being increasingly recognized as a critical component of universal health coverage (UHC), with unmet surgical needs contributing substantially to morbidity, mortality, and financial hardship in low- and middle-income countries. In India, out-of-pocket expenditure (OOPE) remains the dominant mechanism for financing surgical care, raising concerns regarding financial risk protection. Publicly financed health insurance schemes such as the Ayushman Bharat Pradhan Mantri Jan Arogya Yojana (AB-PMJAY) have been introduced to reduce OOPE for inpatient services. Methods: A hospital-based single-center case study with a cross-sectional analytical design was conducted in a tertiary care teaching hospital in coastal Karnataka, India. A total of 150 patients undergoing common general surgical procedures (laparoscopic cholecystectomy, laparoscopic appendicectomy, inguinofemoral hernia repair, and umbilical hernia repair) were enrolled. Patients were categorized into three groups: uninsured, privately insured, and AB-PMJAY beneficiaries. Direct medical expenditure components were captured, and OOPE was compared across groups. Post hoc comparisons were performed following one-way ANOVA. Results: OOPE varied substantially across insurance categories. Uninsured patients incurred the highest mean OOPE, followed by privately insured patients, while AB-PMJAY beneficiaries reported negligible OOPE. Differences across groups were statistically significant (*p* < 0.001). Conclusions: Uninsured patients incurred a high financial burden for common surgical procedures, while private health insurance offered partial financial protection compared to no insurance. AB-PMJAY substantially reduced point-of-care expenditure for eligible beneficiaries. Expanding financial risk protection for surgical care may be essential for advancing equitable access and achieving UHC in India.

## 1. Introduction

Surgery has emerged as a vital component of modern health systems, with an estimated 30% of the global burden of disease requiring timely surgical intervention [1]. Despite this, access to safe, affordable, and timely surgical care remains uneven across low- and middle-income countries (LMICs), where infrastructure, workforce, and financing constraints limit service delivery [2,3,4,5]. The Lancet Commission on Global Surgery highlighted that five billion people worldwide lack access to essential surgical services, and that unmet surgical needs contribute significantly to preventable death and disability [1].

In LMICs, one of the major barriers to accessing surgical care is the high reliance on out-of-pocket expenditure (OOPE), which exposes households to the risk of catastrophic health expenditure (CHE) and impoverishment [6,7]. In India, OOPE accounts for a substantial proportion of total health expenditure, particularly for inpatient and surgical care [8]. Although surgical care is theoretically available across secondary and tertiary hospitals, financial barriers often delay or prevent treatment and may lead to adverse clinical and economic outcomes [9]. From a health systems perspective, out-of-pocket expenditure (OOPE) is best understood as a failure of financial risk protection, one of the core pillars of universal health coverage (UHC). In the context of surgical care—where costs are typically high, episodic, and unpredictable—examining OOPE at the point of service provides a practical lens to assess how different financing mechanisms perform in routine clinical settings.

India has introduced several publicly financed health insurance schemes aimed at improving financial risk protection for inpatient care, the largest being the Ayushman Bharat Pradhan Mantri Jan Arogya Yojana (AB-PMJAY). The scheme provides cashless coverage for inpatient services for eligible households across empanelled public and private hospitals [10]. Private health insurance has also expanded in recent years, although coverage remains limited to a relatively narrow socioeconomic strata, often referred to as the “missing middle”. Consequently, a large share of the population remains uninsured and dependent on OOPE for inpatient surgical care [11,12].

Within this context, examining procedure-level OOPE for common surgical interventions across insurance categories provides a pragmatic way to assess how effectively different financing mechanisms translate into financial risk protection at the point of care. This study presents an analytical single-hospital case study examining OOPE associated with selected general surgical procedures in a tertiary care teaching hospital in coastal Karnataka, and compares expenditure patterns among uninsured individuals, private health insurance beneficiaries, and AB-PMJAY beneficiaries. While not intended to be nationally representative, the case study provides detailed, procedure-level insights relevant to broader discussions on UHC, health financing reforms, and equity in access to surgical care.

## 2. Materials and Methods

This hospital-based single-center case study with a cross-sectional analytical design was conducted in the Department of General Surgery at a tertiary care teaching hospital in coastal Karnataka, India. The study included patients undergoing four common general surgical procedures: laparoscopic cholecystectomy, laparoscopic appendicectomy, inguinofemoral hernia repair, and umbilical hernia repair. These procedures were selected as they represent frequently performed elective surgeries with predictable clinical pathways and comparable perioperative care requirements in tertiary facilities.

A total of 150 patients were enrolled between January and December 2023 using consecutive sampling. Patients aged 18 years and above undergoing any of the four specified procedures were eligible for inclusion. Patients who underwent emergency surgery, had incomplete expenditure data, or declined participation were excluded. For each participant, demographic characteristics, insurance status, and expenditure components were recorded using a structured data collection form. For transparency and reproducibility, the entire dataset analyzed in this study is presented in the Supplementary Materials (Table S1).

Participants were categorized into three mutually exclusive insurance groups: uninsured, privately insured, and beneficiaries of publicly financed insurance under the Ayushman Bharat Pradhan Mantri Jan Arogya Yojana (AB-PMJAY). Uninsured patients financed care through OOPE at the point of service. Privately insured patients utilized their respective private health insurance policies, which typically involved reimbursement-based claims processes and specific coverage limits. AB-PMJAY beneficiaries accessed cashless inpatient services through the hospital’s empanelment under the scheme.

Direct medical expenditure was measured across components common to inpatient surgical care, including diagnostics, pharmacy, consumables, implants (where applicable), procedure-related charges, and bed charges. Expenditure data were obtained from hospital billing records and supplemented with receipts provided by patients and caregivers. OOPE was defined as payments made by households directly at the point of service that were not covered by any insurance mechanism. For AB-PMJAY beneficiaries, expenditure was primarily limited to items not included in the benefit package or to ancillary purchases outside the hospital pharmacy.

Data were analyzed using descriptive and inferential statistics, with the explicit aim of comparing observed OOPE patterns across insurance categories rather than modeling ability to pay or household-level financial coping mechanisms. Mean OOPE across insurance groups was compared using one-way analysis of variance (ANOVA). Post hoc pairwise comparisons were performed to identify statistically significant differences between groups. A *p*-value of < 0.05 was considered statistically significant. All monetary values were recorded in Indian Rupees (INR) and converted to approximate United States Dollar (USD) equivalents for comparative interpretation using the prevailing average exchange rate during the study period [12]. The study is intended as an institutional case study documenting OOPE patterns within a specific hospital setting and is not designed to produce population-level or nationally representative estimates.

### Ethical Considerations

Ethical approval for the study was obtained from the Institutional Ethics Committee of the participating tertiary care teaching hospital. Written informed consent was obtained from all study participants prior to data collection, and confidentiality of patient information was maintained throughout the study in accordance with institutional and national ethical guidelines.

## 3. Results

A total of 150 patients undergoing the four specified general surgical procedures were included in the study. The most common procedure performed was laparoscopic cholecystectomy (40.7%), followed by laparoscopic appendicectomy (30.0%), inguinofemoral hernia repair (18.7%), and umbilical hernia repair (10.7%). The mean age of participants was 44.2 ± 13.6 years, and males constituted 58.0% of the study population. More than half of the patients (56.7%) were beneficiaries of AB-PMJAY, 24.0% had private health insurance, and 19.3% were uninsured. The mean duration of hospitalization was 4.2 ± 1.3 days, with no statistically significant difference across insurance groups (*p* > 0.05).

Marked differences in the magnitude and distribution of OOPE were observed across insurance categories. Uninsured patients incurred the highest OOPE, followed by privately insured patients, while AB-PMJAY beneficiaries incurred negligible OOPE for the specified surgical procedures. The mean total OOPE among uninsured patients was INR 53,420 (≈ USD 640), compared to INR 18,760 (≈ USD 225) among privately insured patients, and INR 1220 (≈USD 15) among AB-PMJAY beneficiaries. One-way ANOVA demonstrated a statistically significant difference in mean OOPE between the three insurance groups (*p* < 0.001). Post hoc pairwise comparisons confirmed that OOPE among uninsured patients was significantly higher compared to privately insured patients (*p* < 0.001) and AB-PMJAY beneficiaries (*p* < 0.001), and that privately insured patients incurred significantly higher OOPE than AB-PMJAY beneficiaries (*p* < 0.001).

When disaggregated by cost components, uninsured patients incurred the highest mean expenditure on diagnostics, consumables, and medicines, while privately insured patients incurred intermediate levels of expenditure across these components due to partial coverage and reimbursement limitations. AB-PMJAY beneficiaries reported negligible expenditure across components due to cashless coverage for inpatient services under the scheme.

Table 1 presents the distribution of 550 cases across the four selected general surgical procedures. Of these, 112 were laparoscopic appendicectomies, 280 laparoscopic cholecystectomies, 81 umbilical hernia repairs, and 77 inguinofemoral hernia repairs.

**Figure 1:**
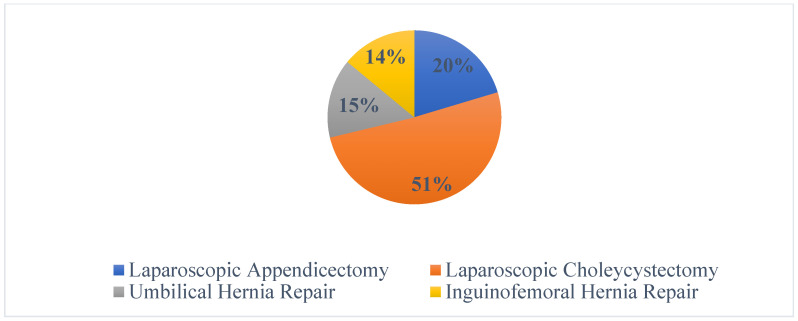
Percentage-wise distribution of surgeries across various categories.

Figure 1 illustrates the percentage distribution of the same 550 procedures. Laparoscopic appendicectomy accounted for 20% of all cases, laparoscopic cholecystectomy for 51%, umbilical hernia repair for 15%, and inguinofemoral hernia repair for 14%.

Table 2 summarizes the number of cases and billing amounts for the four general surgical procedures by insurance category. Among AB-PMJAY beneficiaries, there were 21 laparoscopic appendicectomies, 115 laparoscopic cholecystectomies, 22 umbilical hernia repairs, and 9 inguinofemoral hernia repairs. Among privately insured patients, the corresponding numbers were 37, 58, 25, and 34, while among uninsured patients they were 54, 107, 34, and 34 respectively.

For AB-PMJAY beneficiaries, the median billing amounts for laparoscopic appendicectomy and laparoscopic cholecystectomy were INR 55,917 (≈USD 648.16) and INR 43,061 (≈USD 499.14), and the mean billing amounts for umbilical and inguinofemoral hernia repairs were INR 82,132 (≈USD 952.03) and INR 66,341 (≈USD 768.99). Among privately insured patients, median billing amounts for laparoscopic appendicectomy, laparoscopic cholecystectomy, and inguinofemoral hernia repair were INR 62,936 (≈USD 729.52), INR 53,318 (≈USD 618.04), and INR 92,513 (≈USD 1,072.37), while the mean billing amount for umbilical hernia repair was INR 109,243 (≈USD 1266.29). Among uninsured patients, median billing amounts for laparoscopic appendicectomy, laparoscopic cholecystectomy, and inguinofemoral hernia repair were INR 49,626 (≈USD 575.24), INR 47,673 (≈USD 552.60), and INR 63,085 (≈USD 731.25), and the mean billing amount for umbilical hernia repair was INR 86,837 (≈USD 1006.57).

OOPE as a percentage of the total billing amount was zero across all procedures for AB-PMJAY beneficiaries, between 14.21% and 17.72% for privately insured patients, and 100% for uninsured patients. Median and mean OOPE among AB-PMJAY beneficiaries were zero for all procedures. Among privately insured patients, OOPE ranged from INR 6149 (≈USD 71.28) to INR 10,284 (≈USD 119.21), while among uninsured patients it ranged from INR 47,673 (≈USD 552.60) to INR 86,837 (≈USD 1006.57).

### 3.1. Total Billing of the Selected General Surgeries

**Figure 2:**
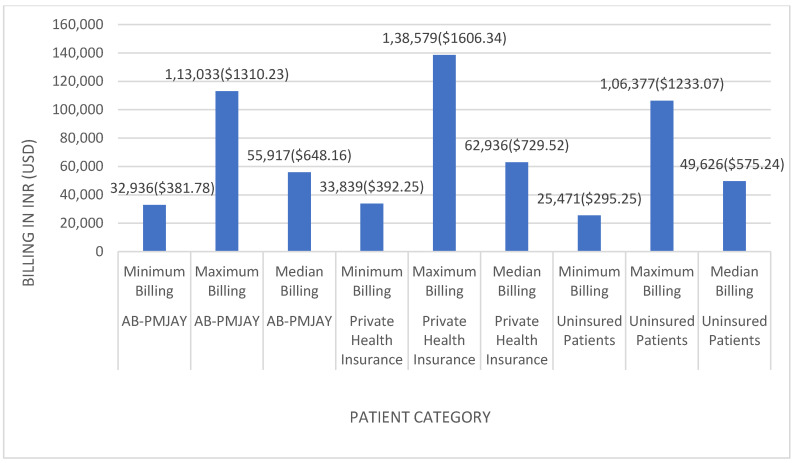
Laparoscopic appendicectomy billing: minimum, maximum and median.

Figure 2 shows the minimum, maximum, and median billing amounts for laparoscopic appendicectomy across the three insurance categories. Among AB-PMJAY beneficiaries, billing ranged from INR 32,936 (≈USD 381.78) to INR 113,033 (≈USD 1310.22), with a median of INR 55,917 (≈USD 648.16). Among privately insured patients, billing ranged from INR 33,839 (≈USD 392.25) to INR 138,579 (≈USD 1,606.34), with a median of INR 62,936 (≈USD 729.52). Among uninsured patients, billing ranged from INR 25,471 (≈USD 295.25) to INR 106,377 (≈USD 1233.07), with a median of INR 49,626 (≈USD 575.24).

Median values were used as the measure of central tendency because the Shapiro–Wilk test yielded *p* < 0.05, indicating non-normal distribution of billing amounts.

Variation in billing amounts across insurance categories may reflect differences in ward type and associated charges. AB-PMJAY beneficiaries are generally allotted general ward beds, while privately insured patients have limited options, and uninsured patients can select bed type according to preference. Total billing, nursing charges, and consultation fees vary accordingly.

**Figure 3:**
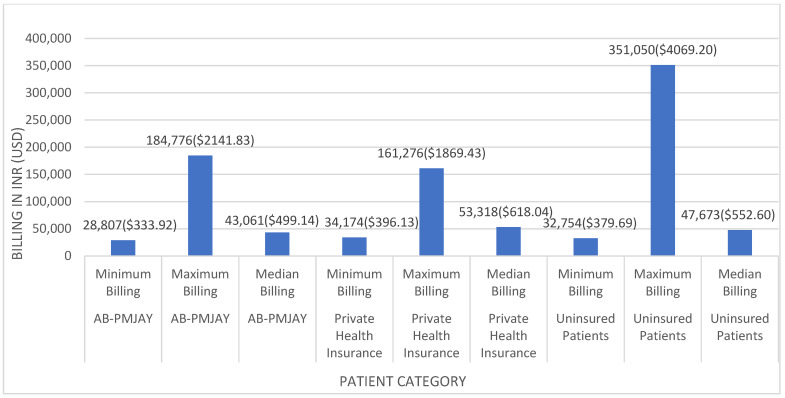
Laparoscopic cholecystectomy billing: minimum, maximum, median and mean.

Figure 3 shows the minimum, maximum, and median billing amounts for laparoscopic cholecystectomy across the three insurance categories. Among AB-PMJAY beneficiaries, billing ranged from INR 28,807 (≈USD 333.92) to INR 184,776 (≈USD 2141.83), with a median of INR 43,061 (≈USD 499.14). Among privately insured patients, billing ranged from INR 34,174 (≈USD 396.13) to INR 161,276 (≈USD 1869.43), with a median of INR 53,318 (≈USD 618.04). Among uninsured patients, billing ranged from INR 32,754 (≈USD 379.67) to INR 351,050 (≈USD 4069.20), with a median of INR 47,673 (≈USD 552.60).

Median values were used due to non-normal distribution of billing amounts (Shapiro–Wilk *p* < 0.05). Variation in billing amounts may reflect differences in ward type and associated charges. AB-PMJAY beneficiaries are generally allotted general ward beds, privately insured patients have limited options, and uninsured patients may select bed type according to preference, with differences in total billing, nursing charges, and consultation fees accordingly.

**Figure 4:**
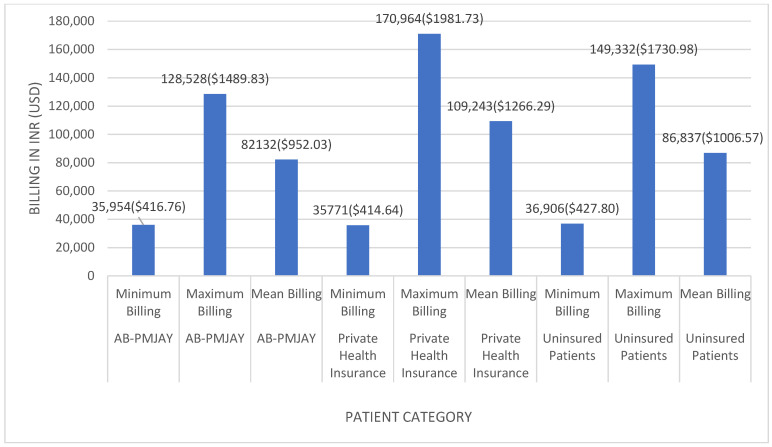
Umbilical hernia repair billing: minimum, maximum and mean.

Figure 4 shows the minimum, maximum, and mean billing amounts for umbilical hernia repair across the three insurance categories. Among AB-PMJAY beneficiaries, billing ranged from INR 35,954 (≈USD 416.76) to INR 128,528 (≈USD 1489.83), with a mean of INR 82,132 (≈USD 952.03). Among privately insured patients, billing ranged from INR 35,771 (≈USD 414.64) to INR 170,964 (≈USD 1981.73), with a mean of INR 109,243 (≈USD 1266.29). Among uninsured patients, billing ranged from INR 36,906 (≈USD 427.80) to INR 149,332 (≈USD 1730.98), with a mean of INR 86,837 (≈USD 1006.57).

Mean values were used as the measure of central tendency due to normal distribution of billing amounts (Shapiro–Wilk *p* > 0.05).

**Figure 5:**
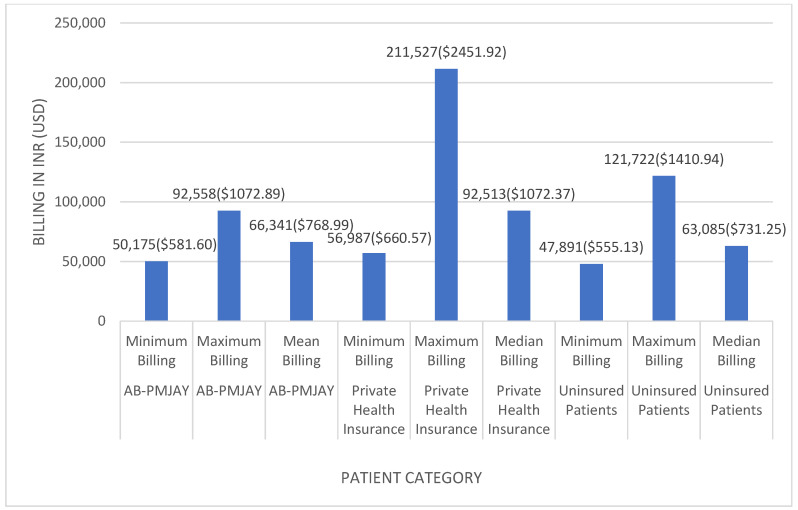
Inguinofemoral hernia repair billing: minimum, maximum, mean and median.

Figure 5 shows the minimum, maximum, and measures of central tendency for inguinofemoral hernia repair across the three insurance categories. Among AB-PMJAY beneficiaries, billing ranged from INR 50,175 (≈USD 581.60) to INR 92,558 (≈USD 1072.89), with a mean of INR 66,341 (≈USD 768.99). Among privately insured patients, billing ranged from INR 56,987 (≈USD 660.57) to INR 211,527 (≈USD 2451.92), with a median of INR 92,513 (≈USD 1072.37). Among uninsured patients, billing ranged from INR 47,891 (≈USD 555.13) to INR 121,722 (≈USD 1410.94), with a median of INR 63,085 (≈USD 731.25).

Median values were used for privately insured and uninsured patients due to non-normal distribution (Shapiro–Wilk *p* < 0.05). Mean values were used for AB-PMJAY beneficiaries due to normally distributed billing data (Shapiro–Wilk *p* > 0.05).

### 3.2. Variation in OOPE Across Patient Categories

Table 3 shows the variation in OOPE for laparoscopic appendicectomy across the three insurance categories. All 21 AB-PMJAY beneficiaries reported zero OOPE due to full cashless coverage. Among uninsured patients (*n* = 54), OOPE was 100%, with a median of INR 49,626 (≈USD 575.24). Among privately insured patients (*n* = 37), OOPE was lower, with an average of 17.62% and a median of INR 7547 (≈USD 87.48).

OOPE varied across private insurers. Medicare Patient, Star Health and Allied Insurance Co. Ltd., Sampoorna Suraksha, and Medi Assist India TPA Pvt. Ltd. reported average OOPEs of 24.76%, 21.77%, 15.74%, and 13.21%, respectively. HDFC, Care Health Insurance Co. Ltd., and Paramount Health Services Insurance TPA Pvt. Ltd. reported average OOPEs of 0%, 6.44%, and 8.74%, respectively. Mean OOPE for Medicare Patient, Star Health and Allied Insurance Co. Ltd., and Medi Assist India TPA Pvt. Ltd. was INR 20,319 (≈USD 235.53), INR 14,370 (≈USD 170.74), and INR 11,230 (≈USD 130.17), respectively. Sampoorna Suraksha had a median OOPE of INR 5,725 (≈USD 66.36). Mean and median OOPE were equal for Care Health Insurance Co. Ltd., Paramount Health Services Insurance TPA Pvt. Ltd., and HDFC at INR 5996 (≈USD 69.50), INR 5390 (≈USD 62.48), and INR 0 (≈USD 0), respectively. HDFC had only one case and was the only private insurer providing zero OOPE for laparoscopic appendicectomy.

Private health insurance providers and third-party administrators (TPAs) included in the analysis were Medicare TPA (New Delhi, India), Star Health and Allied Insurance Co. Ltd. (Chennai, India), Sampoorna Suraksha (Karnataka, India), Medi Assist India TPA Pvt. Ltd. (Bengaluru, India), Care Health Insurance Company Ltd. (Gurugram, India), HDFC ERGO Health Insurance Ltd. (Mumbai, India), Paramount Health Services Insurance TPA Pvt. Ltd. (New Delhi, India), ICICI Lombard General Insurance Company Ltd. (Mumbai, India), Bajaj Allianz General Insurance Company Ltd. (Pune, India), Universal Sompo General Insurance Company Ltd. (Mumbai, India), Vidal Health TPA Pvt. Ltd. (Hyderabad, India), Raksha Health Insurance TPA Pvt. Ltd. (Ahmedabad, India), and MD India Health Insurance TPA Pvt. Ltd. (Pune, India).

**Figure 6:**
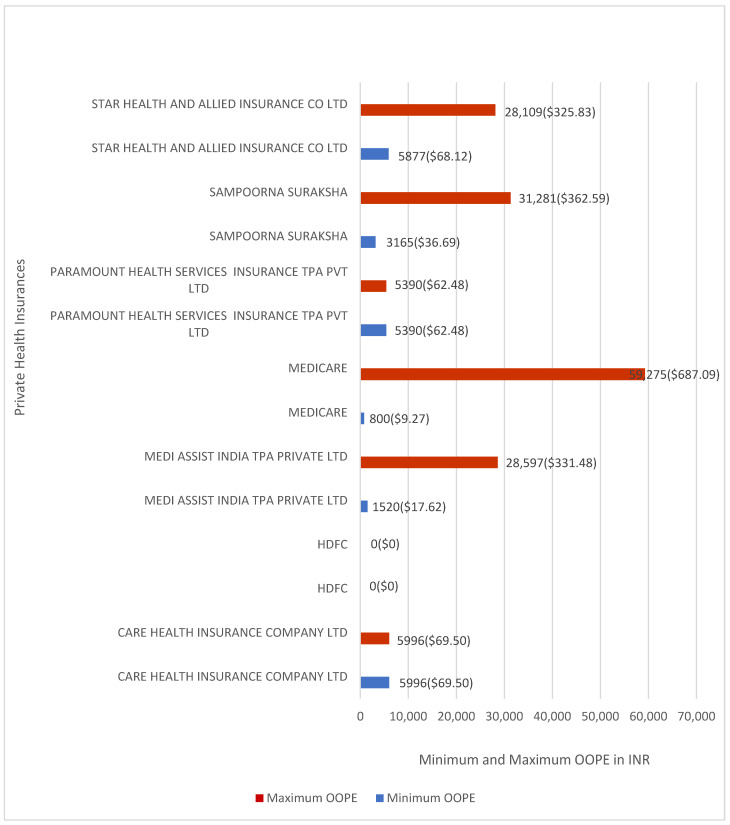
Minimum and maximum OOPE for different private health insurance schemes for laparoscopic appendicectomy.

Figure 6 shows the minimum and maximum OOPE for laparoscopic appendicectomy across private insurance providers. OOPE ranged from INR 800 (≈USD 9.27) to INR 59,275 (≈USD 687.09) for Medicare Patient, from INR 3165 (≈USD 36.69) to INR 31,281 (≈USD 362.59) for Sampoorna Suraksha, and from INR 1520 (≈USD 17.62) to INR 28,597 (≈USD 331.48) for Medi Assist India. For Star Health and Allied Insurance Co. Ltd., OOPE ranged from INR 5,877 (≈USD 68.12) to INR 28,109 (≈USD 325.83). Paramount Health Services Insurance TPA Pvt. Ltd. and Care Health Insurance Co. Ltd. reported OOPE of INR 5390 (≈USD 62.48) and INR 5996 (≈USD 69.50), respectively, with one case each. HDFC had one case and reported zero OOPE.

**Figure 7:**
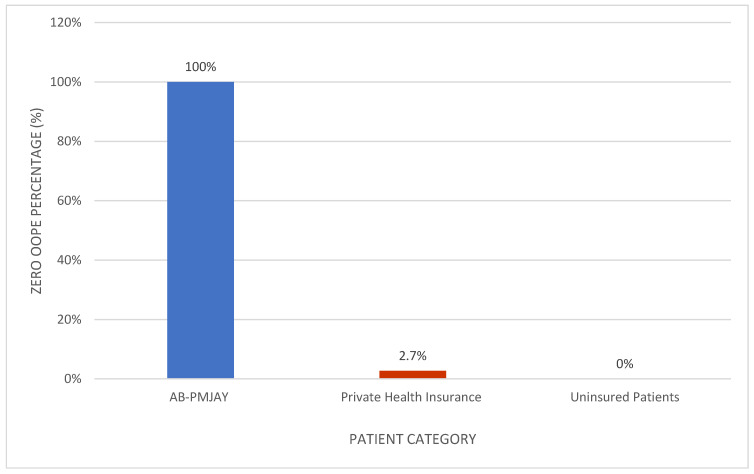
Percentage of laparoscopic appendicectomy patients with zero OOPE across patient category.

Figure 7 shows the proportion of patients with zero OOPE for laparoscopic appendicectomy. All AB-PMJAY beneficiaries (100%) had zero OOPE. Among privately insured patients, 2.7% reported zero OOPE, and none of the uninsured patients had zero OOPE.

Table 4 shows the variation in OOPE for laparoscopic cholecystectomy across the three insurance categories. All AB-PMJAY beneficiaries (*n* = 115) reported zero OOPE due to full cashless coverage. All uninsured patients (*n* = 107) had 100% OOPE, with a median of INR 47,673 (≈USD 552.60). Among privately insured patients, the average OOPE percentage was 15.45% and the median OOPE was INR 6149 (≈USD 71.28).

OOPE varied across private insurers. Average OOPE percentages ranged from 5.09% to 45.26%. Vidal Health TPA Pvt. Ltd. had the highest average OOPE percentage (45.26%) and the highest mean OOPE (INR 62,010; ≈USD 718.79). ICICI Lombard General Insurance and Raksha Health Insurance TPA Pvt. Ltd. reported the lowest average OOPE percentages at 5.09% and 8.72%, respectively. ICICI Lombard General Insurance also reported the lowest mean OOPE at INR 3069 (≈USD 35.57).

**Figure 8:**
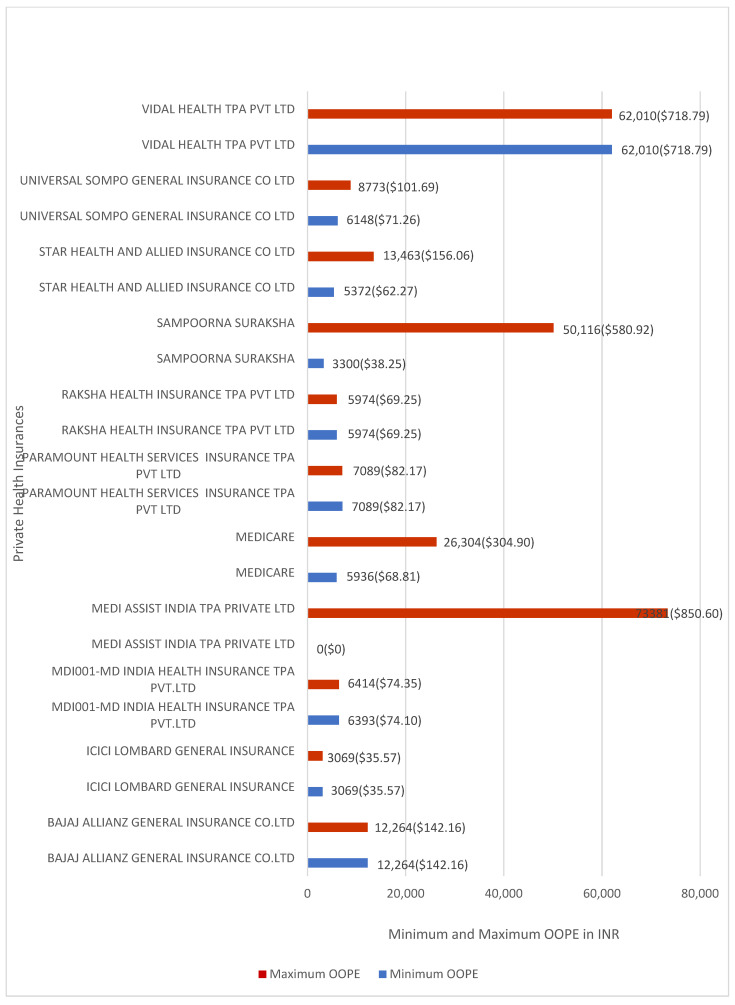
Minimum and maximum OOPE for different private health insurance schemes for laparoscopic cholecystectomy.

Figure 8 shows the minimum and maximum OOPE for laparoscopic cholecystectomy across private insurers. Medi Assist India reported OOPE ranging from INR 0 (≈USD 0) to INR 73,381 (≈USD 881.63). Sampoorna Suraksha reported OOPE ranging from INR 3300 (≈USD 38.25) to INR 50,116 (≈USD 580.92), and Medicare Patient from INR 5936 (≈USD 68.81) to INR 26,304 (≈USD 304.90). Star Health and Allied Insurance Co. Ltd. reported OOPE ranging from INR 5,372 (≈USD 62.27) to INR 13,463 (≈USD 156.06), and Universal Sompo General Insurance Co. Ltd. from INR 6148 (≈USD 71.26) to INR 8773 (≈USD 101.69). MDI001–MD India Health Insurance TPA Pvt. Ltd. reported OOPE ranging from INR 6393 (≈USD 74.10) to INR 6414 (≈USD 74.35). Vidal Health TPA Pvt. Ltd., Raksha Health Insurance TPA Pvt. Ltd., Paramount Health Services Insurance TPA Pvt. Ltd., ICICI Lombard General Insurance, and Bajaj Allianz General Insurance Co. Ltd. had one case each and reported zero OOPE.

**Figure 9:**
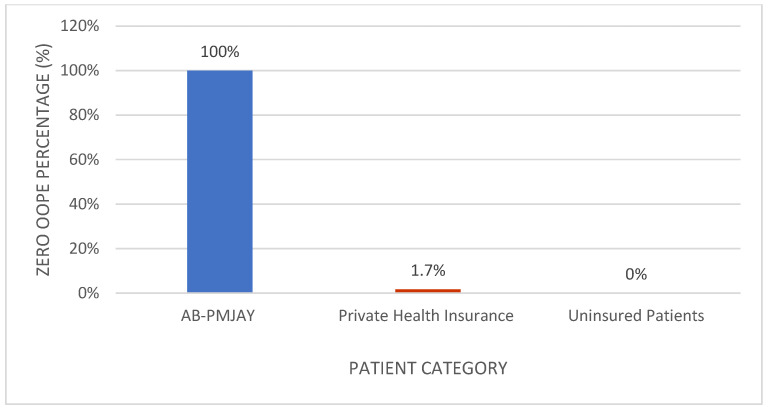
Percentage of laparoscopic cholecystectomy patients with zero OOPE across patient category.

Figure 9 shows the proportion of patients with zero OOPE for laparoscopic cholecystectomy. All AB-PMJAY beneficiaries (100%) had zero OOPE. Among privately insured patients, 1.7% reported zero OOPE, and none of the uninsured patients had zero OOPE.

Table 5 shows the variation in OOPE for umbilical hernia repair across the three insurance categories. All AB-PMJAY beneficiaries (*n* = 22) reported zero OOPE due to full cashless coverage. All uninsured patients (*n* = 34) had 100% OOPE, with a mean of INR 86,837 (≈USD 1006.57). Among privately insured patients, the average OOPE percentage was 14.21% and the median OOPE was INR 10,250 (≈USD 118.81).

OOPE varied across private insurers. Vidal Health TPA Pvt. Ltd. and Medi Assist India TPA Pvt. Ltd. reported the highest average OOPE percentages at 25.40% and 17.54%, respectively, with Vidal Health TPA Pvt. Ltd. recording the highest mean OOPE at INR 35,241 (≈USD 408.50). Raksha Health Insurance TPA Pvt. Ltd. and ICICI Lombard General Insurance reported the lowest average OOPE percentages at 4.54% and 4.79%, respectively, with ICICI Lombard General Insurance reporting the lowest mean OOPE at INR 4772 (≈USD 55.31).

**Figure 10:**
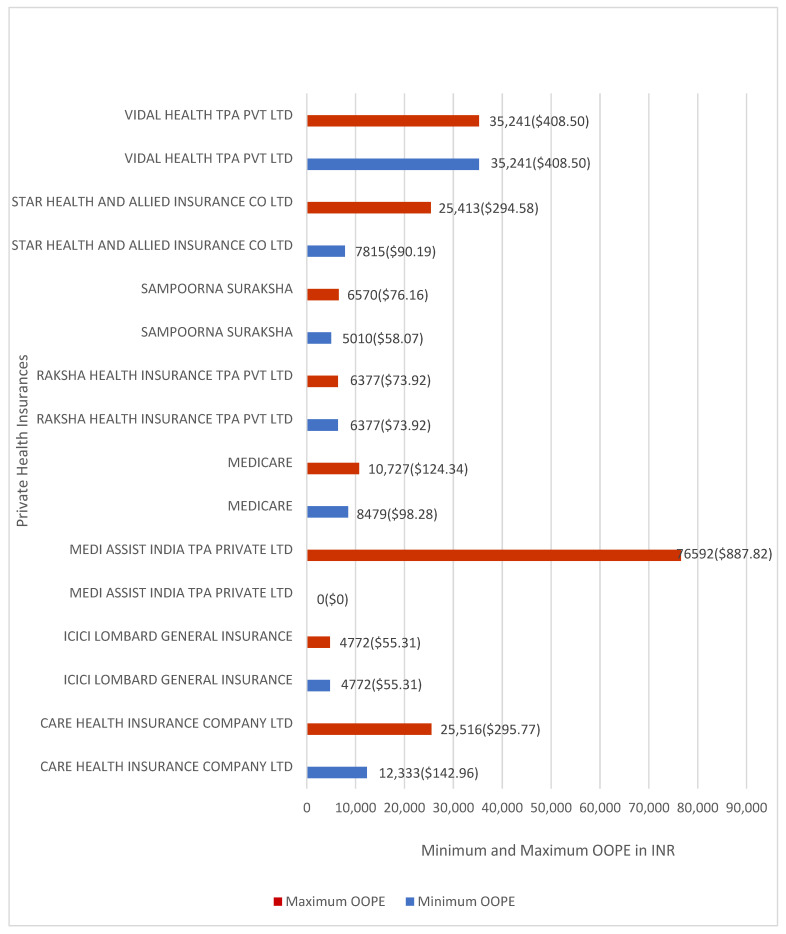
Minimum and maximum OOPE for different private health insurance schemes for umbilical hernia repair.

Figure 10 shows the minimum and maximum OOPE for umbilical hernia repair across private insurers. Medi Assist India reported OOPE ranging from INR 0 (≈USD 0) to INR 76,592 (≈USD 887.82). Star Health and Allied Insurance Co. Ltd. reported OOPE ranging from INR 7815 (≈USD 90.19) to INR 25,413 (≈USD 294.58), and Care Health Insurance Co. Ltd. from INR 12,333 (≈USD 142.96) to INR 25,516 (≈USD 295.77). Sampoorna Suraksha reported OOPE ranging from INR 5,010 (≈USD 58.07) to INR 6,570 (≈USD 76.16), and Medicare Patient from INR 8479 (≈USD 98.28) to INR 10,727 (≈USD 124.34). Vidal Health TPA Pvt. Ltd., Raksha Health Insurance TPA Pvt. Ltd., and ICICI Lombard General Insurance had one case each and reported zero OOPE.

**Figure 11:**
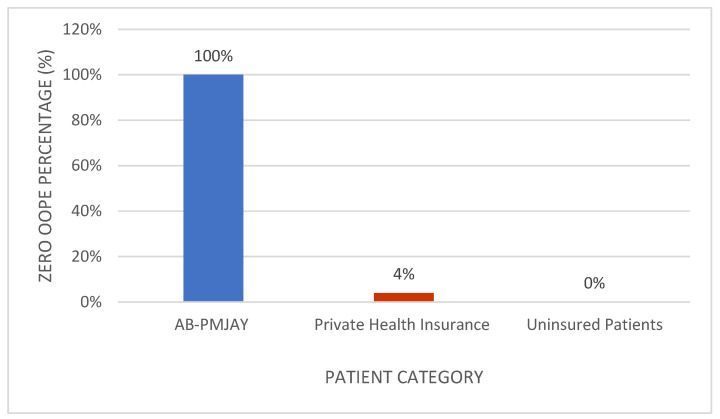
Percentage of umbilical hernia repair patients with zero OOPE across patient category.

Figure 11 shows the proportion of patients with zero OOPE for umbilical hernia repair. All AB-PMJAY beneficiaries (100%) had zero OOPE. Among privately insured patients, 4% reported zero OOPE, and none of the uninsured patients had zero OOPE.

Table 6 shows the variation in OOPE for inguinofemoral hernia repair across the three insurance categories. All AB-PMJAY beneficiaries (n = 9) reported zero OOPE due to full cashless coverage. All uninsured patients (n = 34) had 100% OOPE, with a median of INR 63,085 (≈USD 731.25). Among privately insured patients, the average OOPE percentage was 17.72% and the median OOPE was INR 10,284 (≈USD 119.21).

OOPE varied across private insurers. Average OOPE percentages ranged from 4.03% to 23.37%. Sampoorna Suraksha and Vidal Health TPA Pvt. Ltd. reported the highest average OOPE percentages at 23.37% and 22.73%, respectively, with Vidal Health TPA Pvt. Ltd. recording the highest mean OOPE at INR 21,435 (≈USD 248.46). Raksha Health Insurance TPA Pvt. Ltd. reported the lowest average OOPE percentage at 4.03% and the lowest mean OOPE at INR 5239 (≈USD 60.73).

**Figure 12:**
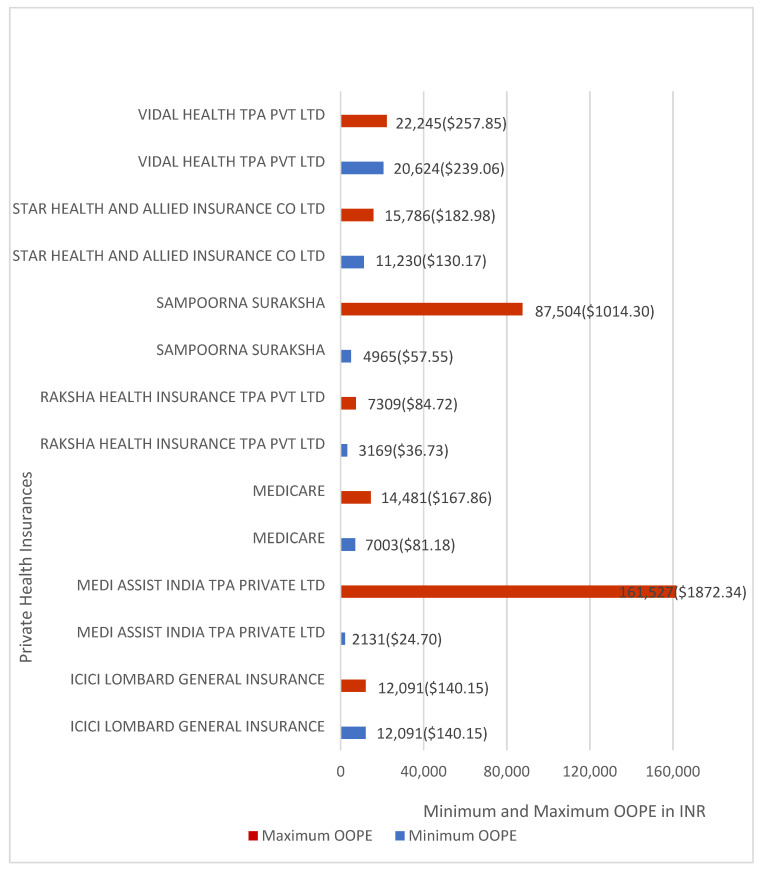
Minimum and maximum OOPE for different private health insurance schemes for inguinofemoral hernia repair.

Figure 12 shows the minimum and maximum OOPE for inguinofemoral hernia repair across private insurers. Medi Assist India reported OOPE ranging from INR 2131 (≈USD 24.70) to INR 161,527 (≈USD 1872.34). Sampoorna Suraksha reported OOPE ranging from INR 4965 (≈USD 57.55) to INR 87,504 (≈USD 1014.30). Medicare Patient reported OOPE ranging from INR 7003 (≈USD 81.18) to INR 14,481 (≈USD 167.86), Star Health and Allied Insurance Co. Ltd. from INR 11,230 (≈USD 130.17) to INR 15,786 (≈USD 182.98), and Raksha Health Insurance TPA Pvt. Ltd. from INR 3,169 (≈USD 36.73) to INR 7309 (≈USD 84.72). Vidal Health TPA Pvt. Ltd. reported OOPE ranging from INR 20,624 (≈USD 239.06) to INR 22,245 (≈USD 257.85). ICICI Lombard General Insurance had one case and reported zero OOPE.

**Figure 13:**
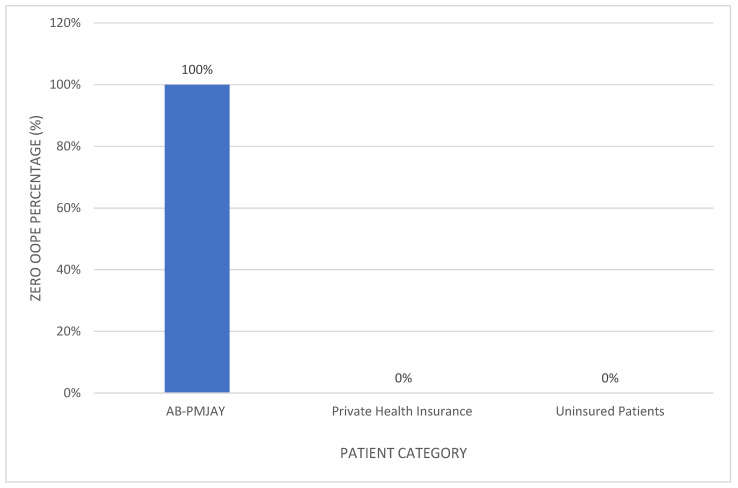
Percentage of inguinofemoral hernia repair patients with zero OOPE across patient category.

Figure 13 shows the proportion of patients with zero OOPE for inguinofemoral hernia repair. All AB-PMJAY beneficiaries (100%) had zero OOPE. No privately insured or uninsured patients had zero OOPE.

### 3.3. Average and Median OOPE for Selected General Surgeries

**Figure 14:**
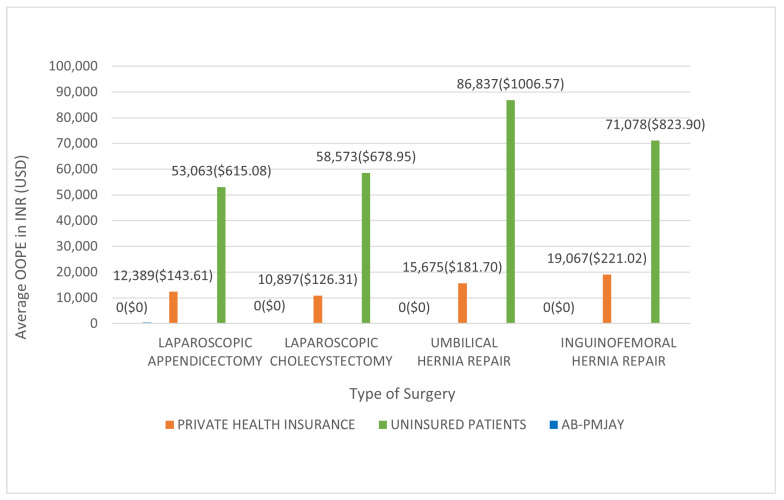
Average OOPE for selected surgeries across patient category.

Figure 14 shows the average OOPE for the four procedures across the three insurance categories. All AB-PMJAY beneficiaries had zero OOPE. Among privately insured patients, average OOPE was INR 12,389 (≈USD 143.61) for laparoscopic appendicectomy, INR 10,897 (≈USD 126.31) for laparoscopic cholecystectomy, INR 15,675 (≈USD 181.70) for umbilical hernia repair, and INR 19,067 (≈USD 221.02) for inguinofemoral hernia repair. Among uninsured patients, the corresponding averages were INR 53,063 (≈USD 615.08), INR 58,573 (≈USD 678.95), INR 86,837 (≈USD 1006.57), and INR 71,078 (≈USD 823.90).

**Figure 15:**
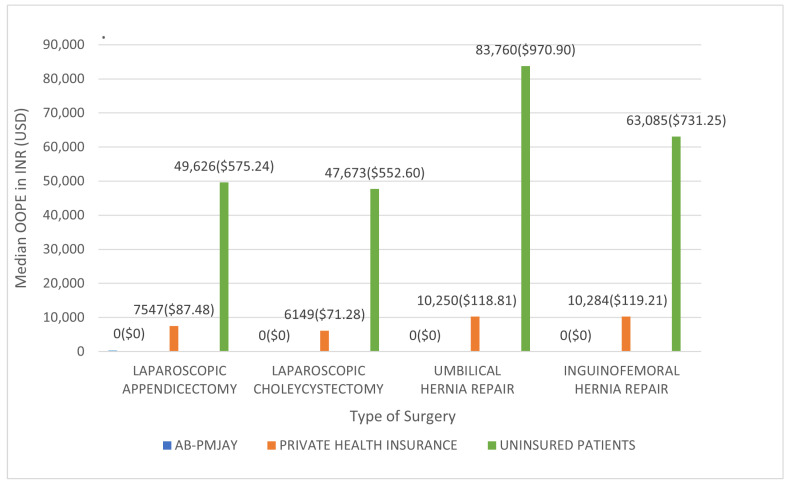
Median OOPE for selected general surgeries across patient category.

Figure 15 shows the median OOPE for the four procedures across the three insurance categories. All AB-PMJAY beneficiaries had zero OOPE. Among privately insured patients, the median OOPE was INR 7547 (≈USD 87.48) for laparoscopic appendicectomy, INR 6149 (≈USD 71.28) for laparoscopic cholecystectomy, INR 10,250 (≈USD 118.81) for umbilical hernia repair, and INR 10,284 (≈USD 119.21) for inguinofemoral hernia repair. Among uninsured patients, the corresponding medians were INR 49,626 (≈USD 575.24), INR 47,673 (≈USD 552.60), INR 83,760 (≈USD 970.91), and INR 63,085 (≈USD 731.25).

## 4. Discussion

While a substantial body of literature exists on out-of-pocket expenditure (OOPE) in India and other low- and middle-income countries, comparatively fewer studies have examined procedure-level OOPE for routine general surgical care with explicit comparison across insurance mechanisms. This single-center case study investigated the OOPE associated with four selected general surgical procedures at a tertiary care teaching hospital in coastal Karnataka, India. The analysis included 550 patients who underwent laparoscopic appendicectomy, laparoscopic cholecystectomy, umbilical hernia repair, and inguinofemoral hernia repair surgeries between January 2023 and December 2023 and were either uninsured or had private health insurance coverage or AB-PMJAY insurance coverage. In 2023, the four most commonly performed general surgical procedures at the study setting were laparoscopic appendicectomy, laparoscopic cholecystectomy, umbilical hernia repair, and inguinofemoral hernia repair surgeries. The number of cases recorded included 112 for laparoscopic appendicectomy, 280 for laparoscopic cholecystectomy, 81 for umbilical hernia repair, and 77 for inguinofemoral hernia repair. Variations in the average/median OOPE were observed in the study across all four selected general surgeries: laparoscopic appendicectomy, laparoscopic cholecystectomy, umbilical hernia repair, and inguinofemoral hernia repair among beneficiaries of AB-PMJAY, patients with private health insurance coverage, and uninsured patients. This study does not attempt to estimate households’ ability to pay, coping strategies, or long-term impoverishment effects associated with surgical OOPE. Such analyses require income, consumption, or asset data and are better addressed through household surveys or longitudinal designs. Instead, the present analysis focuses on point-of-care OOPE as an indicator of how different insurance arrangements perform in protecting patients from immediate financial exposure during hospitalization. As a single-hospital case study, the findings should be interpreted as illustrative rather than representative. However, such case studies play an important role in health systems research by revealing how insurance design, provider payment mechanisms, and hospital billing practices interact in real clinical settings—details that are often obscured in large household surveys or administrative datasets.

The results of this study underscore the significance of the AB-PMJAY scheme in effectively lowering OOPE and enhancing financial risk protection among patients undergoing general surgical procedures. In total, 100% of patients covered under the AB-PMJAY scheme incurred no OOPE across all analyzed general surgical procedures. In contrast, private health insurance schemes lowered OOPE compared to uninsured patients, who experienced the highest OOPE.

As presented in Table 2, the analysis revealed that the average and median OOPEs under the AB-PMJAY scheme were zero across all analyzed general surgeries. Conversely, uninsured patients had substantial median OOPE, ranging from Rs.47,673 (552.60 USD) for laparoscopic cholecystectomy to Rs.63,085 (731.25 USD) for inguinofemoral hernia Repair. Such a high OOPE may cause financial burden and negative health implications [13].

The highest OOPE was experienced by uninsured patients in the study, which is in line with worldwide data showing that uninsured status is a significant contributor to CHE [14]. In particular, our findings highlight the need for policy actions to increase insurance coverage for vulnerable populations who are not covered either under private health insurance schemes or under the AB-PMJAY scheme.

There are multiple explanations for the differences in OOPE between the groups. For AB-PMJAY beneficiaries, the government program’s comprehensive coverage, which fully reimburses medical expenses and relieves patients of any financial strain, explains the zero OOPE. These variations show that although private health insurance can lower OOPE, government programs like AB-PMJAY provide a higher degree of protection [15]. Despite insurance coverage, OOPE remains a challenge. The universal scheme has helped reduce financial burdens, but inequities persist.

The effectiveness of government-financed health insurance coverage schemes in providing monetary security varies [15]. The observed disparities in OOPE among beneficiaries of private health insurance reflect the combined effects of bed classification policies, package exclusions, reimbursement ceilings, and formal or informal co-payment requirements, all of which have implications for equitable access to surgical care. Notably, the median OOPE under private health insurance coverage varied substantially from Rs.6149 (71.28 USD) for laparoscopic cholecystectomy to Rs.10,284 (119.21 USD) for inguinofemoral hernia repair. These results align with earlier findings regarding the limitations of private insurance coverage [16]. Patients covered under private health insurance schemes had lower OOPE than uninsured patients, although they still suffered considerable OOPE. This could be due to policy exclusions, co-payments, or non-covered services. This suggests that, unlike health insurance programs supported by the government, private health insurance plans might not fully cover the complete expense of these general surgical treatments for patients [17,18]. International experience from publicly financed health systems suggests that cashless coverage for essential inpatient services, including surgery, is central to effective financial risk protection, whereas reliance on private insurance alone often results in residual OOPE even among insured populations.

This study provides robust evidence that AB-PMJAY effectively eliminates OOPE for general surgical patients, offering a viable model for India’s universal healthcare coverage. While private health insurance models reduce costs, they do not provide complete financial security, and uninsured patients remain highly vulnerable. Policy efforts should focus on expanding AB-PMJAY, improving private health insurance regulations, and reducing non-medical expenses to achieve equitable healthcare financing.

However, the fact that not all Indians are covered under the AB-PMJAY scheme raises serious issues regarding disparities in access to healthcare [8]. The program is intended to cover the lowest 40% of those who are socioeconomically fragile, leaving the remaining 60% with a sizable gap. Of this uninsured population, only around 30% have private health insurance coverage, and the remaining 30% are uninsured and responsible for paying for all of their medical bills. Because AB-PMJAY beneficiaries have complete inpatient coverage, many others either incur hefty OOPEs or are compelled to postpone critical care, creating glaring disparities in financial protection [8,19].

Lower-and middle-income households are disproportionately impacted by OOPE under ongoing healthcare reforms. These households are more likely to be pushed into poverty, emphasizing the urgent need for expanded public financing and subsidies, particularly for medicines and non-covered services [13].

Achieving equitable access to high-quality healthcare services through universal healthcare coverage is significantly hampered by high OOPE [20]. In order to reduce OOPE and address disparities in general surgical healthcare utilization, it is imperative that government-funded insurance programs like AB-PMJAY be expanded [21]. These initiatives can potentially lower financial obstacles and encourage increased involvement in the healthcare system.

The future research should focus on addressing disparities in healthcare access and outcomes among missing middle populations, ensuring equitable delivery of care.

The findings of this study are limited in their generalizability due to the sample size being limited to a single tertiary care teaching hospital located in a Tier-2 city of coastal Karnataka, India. Larger, more varied sample sizes in future studies may offer a deeper understanding of how OOPE varies by region, healthcare system, and demographics.

## 5. Conclusions

This single-hospital case study offers detailed, real-world perspectives on the financial burden associated with selected general surgical procedures among patients with different insurance statuses at a tertiary care teaching hospital in coastal Karnataka. The study highlights how AB-PMJAY translates policy intent into near-complete financial risk protection for routine general surgical care at the point of service. The findings underscore the need for policy interventions aimed at expanding public insurance coverage and improving the effectiveness of private health insurance in reducing OOPE. Furthermore, there is a compelling need for policy-level interventions to ensure the inclusion of economically vulnerable and uninsured populations. By addressing these disparities, India can move closer to its goal of making surgical care both accessible and equitable for everyone.

### Limitations

This study is not without limitations. The study might be limited to the available data and resources, and incomplete or inconsistent records might restrict comprehensive analysis. The hospital-based design also limits the ability to examine socioeconomic gradients, household coping strategies, or affordability relative to income, which should be addressed in future research integrating clinical and household-level data. The findings of this study are limited in their generalizability due to the sample size being limited to a single tertiary care teaching hospital located in a Tier-2 city of coastal Karnataka, India. Larger, more varied sample sizes in future studies may offer a deeper understanding of how OOPE varies by region, healthcare system, and demographics. Additionally, the study only considered inpatient data from January 2023 to December 2023, focusing solely on costs incurred during the admission-to-discharge period. Only expenditures related to surgical intervention, diagnostics, consumables, and drugs during hospitalization were included, while costs outside this defined window were excluded. This focus might not capture the full spectrum of financial burden faced by patients. Future research with larger, multi-center samples and the inclusion of both direct and indirect costs is necessary for gaining deeper insights regarding financial risk protection in surgical care. It is also important for future studies to explore ways to address disparities in healthcare access and outcomes among missing middle populations, ensuring equitable delivery of care.

## Figures and Tables

**Table 1:** Distribution of surgeries across various categories.

Surgeries	Number of Cases
Laparoscopic Appendicectomy	112
Laparoscopic Cholecystectomy	280
Umbilical Hernia Repair	81
Inguinofemoral Hernia Repair	77
Total	550

**Table 2:** Patient category, type of surgery, number of cases, total percentage (%) of cases, median and mean billing amount, OOPE as average percentage (%) of billing amount, median and mean OOPE.

Patient Category	Type of Surgery	No. of Cases	Total Percentage of Cases (%)	Median Billing Amount/ Mean Billing Amount (INR)	OOPE as Average Percentage (%) of Billing Amount	Median OOPE/ Mean OOPE(INR)
AB-PMJAY	Laparoscopic Appendicectomy	21	3.82	55,917 * ($648.16)	0 ($0)	0 ($0)
	Laparoscopic Cholecystectomy	115	20.91	43,061 * ($499.14)	0 ($0)	0 ($0)
	Umbilical Hernia Repair	22	4.0	82,132 ** ($952.03)	0 ($0)	0 ($0)
	Inguinofemoral Hernia Repair	9	1.64	66,341 ** ($768.99)	0 ($0)	0 ($0)
	Total	167	30.36			
Private Health Insurance	Laparoscopic Appendicectomy	37	6.73	62,936 * ($729.52)	17.62	7547 *** ($87.48)
	Laparoscopic Cholecystectomy	58	10.55	53,318 * ($618.04)	15.45	6149 ***($71.28)
	Umbilical Hernia Repair	25	4.55	109,243 ** ($1266.29)	14.21	10,250 *** ($118.81)
	Inguinofemoral Hernia Repair	34	6.18	92,513 * ($1072.37)	17.72	10,284 *** ($119.21)
	Total	154	28			
Uninsured Patients	Laparoscopic Appendicectomy	54	9.82	49,626 * ($575.24)	100	49,626 *** ($575.24)
	Laparoscopic Cholecystectomy	107	19.45	47,673 * ($552.60)	100	47,673 *** ($552.60)
	Umbilical Hernia Repair	34	6.18	86,837 ** ($1006.57)	100	86,837 **** ($1006.57)
	Inguinofemoral Hernia Repair	34	6.18	63,085 * ($731.25)	100	63,085 *** ($731.25)
	Total	229	41.64			
Grand Total of all Surgeries	550					

* Median billing was considered to measure the central tendency for billing amount where the *p*-value in the Shapiro–Wilk test was <0.05, suggesting a marked divergence from normal distribution. (e.g., laparoscopic appendicectomy under AB-PMJAY). ** Mean billing was considered where the *p*-value was >0.05, indicating a normal distribution (e.g., umbilical hernia repair under AB-PMJAY). *** Median OOPE was considered to measure the central tendency for billing amount where the *p*-value in the Shapiro–Wilk test was <0.05, suggesting a marked divergence from normal distribution. (e.g., laparoscopic appendicectomy under private health insurance). **** Mean OOPE was considered where the *p*-value was >0.05, indicating a normal distribution (e.g., umbilical hernia repair under uninsured patients).

**Table 3:** Variation in OOPE across patient category in laparoscopic appendicectomy.

Patient Category	Number of Cases	Total Percentage (%) of Cases	Average Percentage (%) of OOPE	Median OOPE/Mean OOPE * (INR)
AB-PMJAY	21	3.81	0	0 ($0)
Uninsured patients	54	9.82	100	49,626 *** ($575.24)
Private Health Insurances	37	6.73	17.62	7547 *** ($87.48)
Sampoorna Suraksha	15	2.73	15.74	5725 * ($66.36)
Medicare	10	1.82	24.76	20,319 **** ($235.53)
Medi assist India TPA Pvt. Ltd.	05	0.91	13.21	11,230 **** ($130.17)
Star Health and Allied Insurance Co. Ltd.	04	0.73	21.77	14,730 **** ($170.74)
Care Health Insurance Company Ltd.	01	0.18	6.44	5996 * ($69.50)
HDFC	01	0.18	0	0 ($0)
Paramount health services insurance TPA Pvt. Ltd.	01	0.18	8.74	5390 * ($62.48)

* Median and Mean OOPE in INR (e.g., Care Health Insurance Company Ltd., Paramount Health Services Insurance TPA Pvt. Ltd.). *** Median OOPE was considered to measure the central tendency when the *p*-value was <0.05 in the Shapiro–Wilk test, suggesting a marked divergence from normal distribution. (e.g., Sampoorna Suraksha). **** Mean OOPE was considered when the *p*-value was >0.05 in the Shapiro–Wilk test, indicating a normal distribution (e.g., Medi Assist India TPA Pvt. Ltd., Medicare, Paramount Health Services Insurance TPA Pvt. Ltd., Star Health and Allied Insurance Co. Ltd.).

**Table 4:** Variation in OOPE across patient category in laparoscopic cholecystectomy.

Patient Category	Number of Cases	Total Percentage (%) of Cases	Average Percentage (%) of OOPE	Median OOPE/ Mean OOPE * (INR)
AB-PMJAY	115	20.91	0	0 ($0)
Uninsured patients	107	19.45	100	47,673 *** ($552.60)
Private Health Insurances	58	10.55	15.45	6149 *** ($71.28)
Sampoorna Suraksha	24	4.36	16.84	5063 *** ($58.69)
Medi Assist India TPA Private Ltd.	15	2.73	15.17	7496 *** ($86.89)
Medicare	05	0.91	15.56	13,638 **** ($158.08)
Star Health and Allied Insurance	05	0.91	10.25	8685 **** ($100.67)
MDI001 -MD India Health Insurance TPA Pvt. Ltd.	02	0.36	13.14	6404 * ($74.23)
Universal Sompo general insurance Co. Ltd.	02	0.36	13.25	7461 * ($86.48)
Bajaj Allianz general insurance Co. Ltd.	01	0.18	12.78	12,264 * ($142.16)
ICICI Lombard general insurance	01	0.18	5.09	3069 * ($35.57)
Paramount health service insurance TPA Pvt. Ltd.	01	0.18	10.91	7089 * ($82.17)
Raksha Health Insurance TPA Pvt. Ltd.	01	0.18	8.72	5974 * ($69.25)
Vidal Health TPA Pvt. Ltd.	01	0.18	45.26	62,010 * ($718.79)

* Median and Mean OOPE in INR (e.g., Bajaj Allianz general insurance Co. Ltd., ICICI Lombard general insurance, etc.). *** Median OOPE was considered to measure the central tendency when the *p*-value was <0.05 in the Shapiro–Wilk test, suggesting a marked divergence from a normal distribution. (e.g., Medi Assist India TPA Private Ltd., Sampoorna Suraksha). **** Mean OOPE was considered when the *p*-value was >0.05 in the Shapiro–Wilk test, indicating a normal distribution (e.g., Medicare, Star Health, and Allied Insurance).

**Table 5:** Variation in OOPE across patient category in umbilical hernia repair.

Patient Category	Number of Cases	Total Percentage (%) of Cases	Average Percentage (%) of OOPE	Median OOPE/ Mean OOPE * (INR)
AB-PMJAY	22	4	0	0 ($0)
Uninsured patients	34	6.18	100	86,837 **** ($1006.57)
Private Health Insurances	25	4.55	14.21	10,250 *** ($118.81)
Medi Assist India TPA Private Ltd.	09	1.64	17.54	5469 *** ($63.39)
Star Health and Allied Insurance	05	0.91	13.61	16,641 **** ($192.89)
Medicare	03	0.55	9.89	9819 **** ($113.82)
Sampoorna Suraksha	03	0.55	10.97	5613 **** ($65.06)
Care Health Insurance Company Ltd.	02	0.36	16.02	18,925 * ($219.37)
ICICI Lombard general insurance	01	0.18	4.79	4772 * ($55.31)
Raksha Health Insurance TPA Pvt. Ltd.	01	0.18	4.54	6377 * ($73.92)
Vidal Health TPA Pvt. Ltd.	01	0.18	25.40	35,241 * ($408.50)

* Median and Mean OOPE in INR (e.g., Care Health Insurance Company Ltd., ICICI Lombard general insurance, Raksha Health Insurance TPA Pvt. Ltd., Vidal Health TPA Pvt. Ltd.). *** Median OOPE was considered to measure the central tendency when the *p*-value was <0.05 in the Shapiro–Wilk test, suggesting a marked divergence from a normal distribution. (e.g., Medi Assist India TPA Private Ltd.). **** Mean OOPE was considered when the *p*-value was >0.05 in the Shapiro–Wilk test, indicating a normal distribution (e.g., Medicare, Sampoorna Suraksha, and Star Health and Allied Insurance).

**Table 6:** Variation in OOPE across patient category in inguinofemoral hernia repair.

Patient Category	Number of Cases	Total Percentage (%) of Cases	Average Percentage (%) of OOPE	Median OOPE/ Mean OOPE * (INR)
AB-PMJAY	9	1.64	0	0 ($0)
Uninsured patients	34	6.18	100	63,085 *** ($731.25)
Private Health Insurances	34	6.18	17.72	10,284 *** ($119.21)
Medi Assist India TPA Private Ltd.	10	1.82	20.26	10,797 *** ($125.15)
Sampoorna Suraksha	09	1.64	23.37	6600 *** ($76.50)
Medicare	08	1.45	10.88	11,167 **** ($129.44)
Raksha Health Insurance TPA Pvt. Ltd.	02	0.36	4.03	5239 * ($60.73)
Star Health and Allied Insurance	02	0.36	17.07	13,508 * ($156.58)
Vidal Health TPA Pvt. Ltd.	02	0.36	22.73	21,435 * ($248.46)
ICICI Lombard general insurance	01	0.18	14.91	12,091 * ($140.15)

* Median and mean OOPE in INR (e.g., ICICI Lombard general insurance, Raksha Health Insurance TPA Pvt. Ltd., Star Health and Allied Insurance, Vidal Health TPA Pvt. Ltd.). *** Median OOPE was considered to measure the central tendency when the *p*-value was <0.05 in the Shapiro–Wilk test, suggesting a marked divergence from a normal distribution. (e.g., Medi Assist India TPA Private Ltd. and Sampoorna Suraksha). **** Mean OOPE was considered when the *p*-value was >0.05 in the Shapiro–Wilk test, indicating a normal distribution (e.g., Medicare).

## Data Availability

Data is contained within the article or Supplementary Materials. The original contributions presented in this study are included in the article/Supplementary Materials. Further inquiries can be directed to the corresponding authors.

## Supplementary Materials

The following supporting information can be downloaded at: https://www.mdpi.com/article/10.3390/healthcare14050587/s1, Table S1: Raw Data.
